# A comparison of freezing-damage during isochoric and isobaric freezing of the potato

**DOI:** 10.7717/peerj.3322

**Published:** 2017-05-18

**Authors:** Chenang Lyu, Gabriel Nastase, Gideon Ukpai, Alexandru Serban, Boris Rubinsky

**Affiliations:** 1 Department of Mechanical Engineering, University of California Berkeley, Berkeley, CA, USA; 2 College of Biosystems Engineering and Food Science, Zhejiang University, Hangzhou, Zhejiang, China; 3 Department of Building Services, Transilvania University of Brasov, Brasov, Romania

**Keywords:** Refrigeration, Isochoric, Potato, Preservation, Food, Isobaric

## Abstract

**Background:**

Freezing is commonly used for food preservation. It is usually done under constant atmospheric pressure (isobaric). While extending the life of the produce, isobaric freezing has detrimental effects. It causes loss of food weight and changes in food quality. Using thermodynamic analysis, we have developed a theoretical model of the process of freezing in a constant volume system (isochoric). The mathematical model suggests that the detrimental effects associated with isobaric freezing may be reduced in an isochoric freezing system. To explore this hypothesis, we performed a preliminary study on the isochoric freezing of a produce with which our group has experience, the potato.

**Method:**

Experiments were performed in an isochoric freezing device we designed. The device is robust and has no moving parts. For comparison, we used a geometrically identical isobaric freezing device. Following freezing and thawing, the samples were weighed, examined with colorimetry, and examined with microscopy.

**Results:**

It was found that potatoes frozen to −5 °C in an isochoric system experienced no weight loss and limited enzymatic browning. In contrast the −5 °C isobaric frozen potato experienced substantial weight loss and substantial enzymatic browning. Microscopic analysis shows that the structural integrity of the potato is maintained after freezing in the isochoric system and impaired after freezing in the isobaric system.

**Discussion:**

Tissue damage during isobaric freezing is caused by the increase in extracellular osmolality and the mechanical damage by ice crystals. Our thermodynamic analysis predicts that during isochoric freezing the intracellular osmolality remains comparable to the extracellular osmolality and that isochoric systems can be designed to eliminate the mechanical damage by ice. The results of this preliminary study seem to confirm the theoretical predictions.

**Conclusion:**

This is a preliminary exploratory study on isochoric freezing of food. We have shown that the quality of a food product preserved by isochoric freezing is better than the quality of food preserved to the same temperature in isobaric conditions. Obviously, more extensive research remains to be done to extend this study to lower freezing temperatures and other food items.

## Introduction

This study was designed to compare the damage to a food item, the potato, due to freezing in an isobaric (constant pressure) atmospheric system with the damage due to freezing in an isochoric (constant volume) system. Refrigeration and freezing are one of the most popular methods of food preservation (Mollett, 1996; Tressler & Evers, 1957). Metabolism is temperature dependent and low temperatures aid preservation by reducing deleterious chemical reactions in food and inhibiting the growth of microorganisms and other pathogens. The lower the temperature, the further chemical reactions rates are reduced, and preservation improved. Below the freezing temperature of water, most foods, many of which consist of water, freeze. Freezing, while reducing metabolism and even sometimes killing pathogens, has a number of detrimental effects on food (Reid, 1996). The process of freezing itself will induce several mechanisms of damage. A major mechanism is the “solute-concentration damage.” Freezing removes water from the solutions in the form of ice. Since ice has a tight crystallographic structure and cannot contain any solutes (Rubinsky, 1983a, 1983b), the concentration of the solutes in the unfrozen portion increases with freezing to lower temperatures. The detrimental effects of the increased concentration of solutes can be lumped together under the heading “solute-concentration damage” (Reid, 1996). A mechanism related to solute-concentration damage is “dehydration damage” (Reid, 1996). Biological matter made of cells freezes preferentially an extracellularly (Mazur, 1970; Rubinsky et al., 1987). As a result of the increase in concentration in the extracellular medium, there is an osmotically driven transport of water across the cell membrane, from the interior of the cell to the extracellular medium (Rubinsky et al., 1987). This causes cell dehydration and cell membrane deformation and the so-called “dehydration damage” (Reid, 1996). There are also two mechanisms of mechanical damage. One is mechanical damage from ice crystals, associated with local stress when rigid ice crystals compress or deform organic structure entrapped between the ice crystals (Reid, 1996). The compression is due to the freezing of water in pockets of high concentration solution entrapped between ice crystals (Ishiguro & Rubinsky, 1994). A second is the “ratchet mechanism of damage,” caused by ice growing in the deformed region of tissue in such a way that it prevents the return of the structure to its original shape (Reid, 1996). Damage also occurs during storage of frozen biological matter. However, in this study, we will address only the mechanisms of damage associated with the process of freezing and explore the hypothesis that isochoric preservation can reduce freezing-damage to food, relative to isobaric freezing.

**Figure 1:**
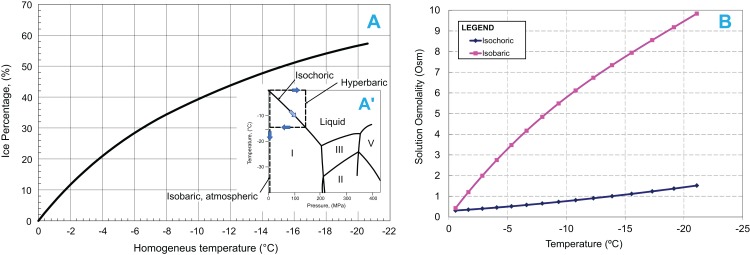
(A) Ice percentage-homogenized temperature diagram during freezing in an isochoric system. The temperature is homogenized with the temperature at which the tested solution freezes at atmospheric pressure. Insert in A′: Pressure–Temperature diagram for water and process lines for isobaric atmospheric, hyperbaric and isochoric freezing processes. (B) Comparison of osmolality as a function of temperature during isochoric and isobaric (at 1 atm) freezing of the same solution.

We have published two fundamental thermodynamic studies on the process of freezing in an isochoric system (Rubinsky, Perez & Carlson, 2005) and on the process of nucleation, supercooling, and vitrification in an isochoric system (Szobota & Rubinsky, 2006). Figure 1 shows the insight gained from the thermodynamic analysis (Rubinsky, Perez & Carlson, 2005). The insert in Fig. 1A, shows the thermodynamic path of a freezing process in an isochoric (constant volume) system, in comparison to the thermodynamic path in an atmospheric isobaric system and in a hyperbaric system. The process of freezing in an atmospheric isobaric system occurs along the vertical line on the phase diagram. In contrast, the process of freezing in a constant volume system occurs along the liquidus line, to the triple point between ice I, ice III, and water. For pure water, the pressure and temperature at the triple point are −21.985 °C and 209.9 MPa, respectively. Under isobaric conditions, the entire amount of water in the system will be frozen at the triple point temperature. The extent of freezing in an isochoric system is different. Figure 1A was obtained from the theoretical analysis (Rubinsky, Perez & Carlson, 2005). It shows the amount of ice in an isochoric system as a function of a homogenized temperature, *T*_H_. *T*_H_ is defined as (*T*_H_ = *T*−*T*_0_) where, *T* is the actual temperature of the isochoric system and *T*_0_ is the temperature at which the solution in the isochoric system freezes at atmospheric pressure. We are using this homogenization, because it makes the curve useful for a wide range of compositions. Thermodynamic analysis predicts that in isochoric freezing, 45% of the water in the system will remain unfrozen at the triple point (Fig. 1A) (Rubinsky, Perez & Carlson, 2005). This suggests that “mechanical damage from freezing” (Reid, 1996) can be eliminated during freezing to temperatures at (or above) the triple point, by designing an isochoric freezing device in which the stored food product occupies the 45% unfrozen volume. Figure 1B shows another interesting observation gained from the thermodynamic analysis of the processes of freezing in an isochoric system and in an isobaric system (Rubinsky, Perez & Carlson, 2005). The figure shows the osmolality of the freezing solution as a function of temperature. The initial osmolality of the solution used in this study was about 0.3086 Osm. Figure 1B shows that in a solution frozen in an isobaric system (atmospheric or hyperbaric), the osmolality at the triple point is more than twenty times as high as the initial osmolality. Obviously, this leads to the “solute-concentration damage” and “dehydration damage” in conventional isobaric freezing (Reid, 1996). In contrast, Fig. 1B shows that in isochoric freezing, the osmolality at the triple point is lower by a factor of five from that during isobaric freezing. This result is to be expected, from the data displayed in Fig. 1A. In isochoric freezing, 45% of the solution remains unfrozen at the triple point. Our results suggest that in addition to eliminating mechanical damage, isochoric freezing will also reduce solute-concentration damage and dehydration damage.

There are additional aspects of isochoric freezing that deserve attention. Nucleation theory predicts that “homogeneous nucleation” (i.e., nucleation without a preferential nucleation site) in pure water is about −42 °C. In pure water without nucleation sites, the water can supercool to −42 °C and freeze at that temperature, rather than at the thermodynamic equilibrium value of 0 °C. In contrast, we have shown from a thermodynamic analysis of the probability of homogeneous nucleation in an isochoric system, that the temperature for “homogeneous nucleation” in water is substantially depressed from about −42 °C in an isobaric atmospheric system to well below −100 °C in an isochoric system (Szobota & Rubinsky, 2006). This suggests that isochoric cooling will promote vitrification, as the homogeneous nucleation temperature in an isochoric system is in a range comparable to the glass formation temperature for water, about −130 °C. The predicted effect of isochoric freezing on nucleation was examined experimentally (Preciado, 2007; Preciado & Rubinsky, 2010). This effect of isochoric freezing was utilized for cryofixation for electron microscopy (Leunissen & Yi, 2009). The predicted effect of isochoric cooling on vitrification (Szobota & Rubinsky, 2006) was shown experimentally (Huebinger, Han & Grabenbauer, 2016).

Isochoric freezing, while maintaining a constant volume, causes an increase in pressure, to the triple point. Freezing of biological matter under elevated pressure was studied in the past (Kalichevsky, Knorr & Lillford, 1995; Persidsky, 1971; LeBail et al., 2002; Toepfl et al., 2006). Hyperbaric freezing (see insert in Fig. 1A) is used for rapid freezing; by first increasing the pressure, followed by cooling to the intersection of the hyperbaric line with the liquidus curve and then decreasing the pressure to atmospheric (LeBail et al., 2002). Treatment of food at hyperbaric pressures (without freezing) is also used for sterilization. Pressure has an effect on microorganisms’ sterilization and complete *Escherichia coli* death was reported at 210 MPa (Suppes et al., 2003). In contrast, the same study found that red blood cells survive this pressure and are more resilient to hyperbaric pressures than *E. coli* (Suppes et al., 2003). Salinas-Almaguer et al. (2015) investigated the use of the increase in pressure caused by isochoric freezing, for sterilization of *E. coli*. In an isochoric system in which the biological material was dispersed throughout the freezing sample, Salinas-Almaguer et al. found that the *E. coli* were completely destroyed at −15 °C, but survived partially at −20 and −30 °C. It should be mentioned that we obtained different results in our isochoric freezing experiments (Preciado, 2007, pp. 81–133). Using three different types of cells, Madin–Darby canine kidney epithelial cells, *E. coli*, and yeast, we found substantial survival after isochoric freezing to −10, −15, and −20 °C. However, as discussed in the previous paragraph, in our experiment the isochoric chamber was designed in a different way. Ice nucleation was induced in such a way as to ensure that the cells reside in the unfrozen volume and there is no contact between the cells and ice crystals. This is how we avoid “mechanical damage from freezing” (Reid, 1996). In the Salinas-Almaguer et al. experiments, the biological material was not separated from the ice crystals. This may explain the difference in our findings. In the study reported here, we have used the same isochoric chamber configuration as in the work of Preciado (2007).

As mentioned earlier, our previous studies (Rubinsky, Perez & Carlson, 2005) suggest that isochoric freezing has the potential to reduce the damage due to freezing in food products, relative to isobaric freezing. The goal of this study is to assess the damages induced by freezing a food product (a potato) in an isochoric system in comparison to the freezing damages in an isobaric system. We have chosen to study isochoric freezing in a potato because we have extensive experience with its mechanisms of damage (Ivorra, Mir & Rubinsky, 2009; Golberg, Rabinowitch & Rubinsky, 2010; Hjouj & Rubinsky, 2010). This study is preliminary and the focus is on freezing-damage in a narrow range of parameters. Our primary goal is a first order exploration of the concept. Obviously, much more work remains to be done to evaluate the effect of isochoric freezing on various food products and to explore the value of isochoric preservation to the food industry.

## Materials and Methods

### Isochoric system

**Figure 2:**
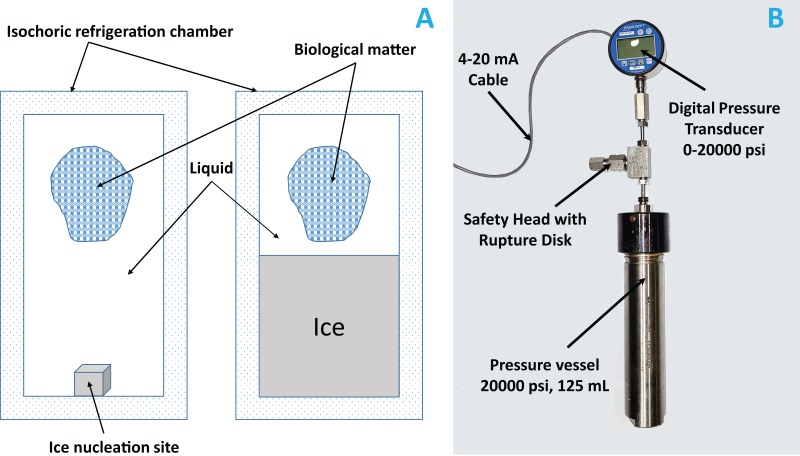
(A) Schematic of an isochoric system; ice nucleation site is the place where we placed a small metal whose role is to initiate ice formation. (B) Photograph of the isochoric system; the height of the reactor without the fittings and the measurements instruments is 10″ and with them is 19″. The inner chamber diameter is 1″.

Isochoric freezing systems are simple. They require only a constant volume chamber, capable of withstanding the pressures that develop in the system, with minimal deformation. For control, they require a pressure transducer. A photograph of the system is shown in Fig. 2A. The isochoric chamber is based on a modified stainless steel OC-1 pressure vessel (O-ring 316 SS, inner cylindrical volume of 125 ml, 1″ inner diameter, working pressure 13,800 psi, test pressure 20,000 psi) custom designed by High Pressure Equipment Company (Erie, PA, USA). We used a standard O-ring made of BUNA-N, for sealing. The constant volume chamber is sealed with a screw and metal seal and is connected to an Ashcroft 4–20 mA loop-powered 20,000 psi pressure gauge, connected through a NI myDAQ Connector (National Instruments, Austin, TX, USA) to a laptop. The data is recorded and displayed with LabVIEW. For safety, a rupture disk limited the pressure to 60 MPa (about 8,700 psi). The isochoric chamber was immersed in a water–ethylene glycol bath (50/50) cooled by means of a NesLab RT-140 cooling system (Thermo Scientific, Waltham, MA, USA).

### Sample preparation

Russian Banana Fingerling potatoes (*Solanum tuberosum* L.) weighing between 12 and 20 g, purchased at a local store, were used in this study. The osmolality of the potatoes was determined in preliminary experiments by measuring the samples’ weight loss in different sucrose solution. We found that a solution of 9.09% w/w (0.3086 Osm) sucrose was isotonic with the potatoes. In preparation for the experiments the samples were peeled, cut into cuboid, weighed, and enclosed into cryogenic vials (standard 12 mm inner diameter, 1.2 ml, Corning Incorporated cryogenic vial, capped and self-standing) filled with the isotonic sucrose solution (9.09% w/w) in such a way to ensure there was no air in the vials. We made a small hole (0.5 mm) in the vial’ wall to ensure thermodynamic and osmolality equilibrium between the interior of the vial and the interior of the isochoric chamber.

### Experimental protocol

The potato samples were divided into three groups: room temperature preservation, isochoric freezing, and isobaric freezing. The room temperature preservation samples were preserved in an isotonic sucrose solution at room temperature (22 °C) for 120 min. The isochoric samples were processed using the isochoric experimental system. A steel nut (the ice nucleating surface) was dropped to the bottom of the isochoric chamber to ensure that ice formation started at the bottom of the chamber at a distance from the vials, which were on the top of the chamber. The isochoric chamber was filled with isotonic sucrose solution and sealed, with care to avoid the entrapment of air bubbles. It is important to emphasize that care must be exercised to eliminate air from the system. The presence of undissolved air can affect the results (Perez et al., 2016). The chamber was then completely immersed in the cooling bath and cooled to −5 °C. We performed the experiments at this temperature, because, in a previous study we found that organisms can survive isochoric freezing at these conditions (Mikus et al., 2016). In the previous study (Mikus et al., 2016), −2, −4, and −6 °C was studied to evaluate whether the organisms can survive isochoric freezing. As the survival rate at −4 and −6 °C was 97% and 71%, respectively, we choose moderate temperature (−5 °C) as our experiment conditions. Obviously, in the future, studies need to be performed in the entire range of temperatures to the triple point. The pressure was monitored and recorded in real time, using LabVIEW. It took about 60 min to reach the desired pressure and the experiment was terminated after another 60 min. The isochoric chamber was warmed at room temperature until the pressure reached atmospheric. Then the chamber was opened for sample analysis. The isobaric samples followed the same procedure as the isochoric samples except that the chamber was open to atmospheric pressure during freezing. The samples were kept in the cooling bath at −5 °C for 120 min. We limited the period of exposure to subfreezing temperatures to 2 h, because our focus is on freezing-damage not the damage due to storage.

It is important to notice the special mode in which ice forms in our system. As indicated in the “Introduction,” our isochoric system is designed in such a way as to separate between the ice and the biological material. We design the volume of the biological material to be less than the volume of the unfrozen fraction at the temperature of interest. At −5 °C, we induce ice nucleation at the bottom of the chamber and the sample is kept at the top of the chamber, in the 75% of the volume that is unfrozen. In this way, we eliminate the possibility of mechanical damage by freezing (Reid, 1996).

### Sample analysis

Three methods were used to evaluate and compare the samples preserved at room temperature to isobarically frozen samples and isochorically preserved samples: weight loss, color change, and microscopic appearance. The samples were analyzed immediately after removal from the chambers.

To evaluate weight loss, the samples were weighed before and immediately after each treatment with an electronic balance (ER-182A; A&D Company, Tokyo, Japan). The surface water on the sample was absorbed by filter papers before weighing. This was repeated for five samples, for each treatment.

Colorimetric measurements were done with a color meter (TES-135A; TES Electric Electronic Corp., Taiwan) in Hunter L* a* b* color space values before and immediately after each treatment. The L*, a*, b* represent the lightness of the color (L* = 0 yields black and L* = 100 indicates diffuse white; specular white may be higher), the redness of the color (a*, negative values indicate green while positive values indicate magenta), and the yellowness of the color (b*, negative values indicate blue and positive values indicate yellow).

The total color difference (Δ*E*) between the differently treated samples was calculated as follows (Cserhalmi et al., 2006):

$$\Delta E = \sqrt {\Delta {L^2} + \Delta {a^2} + \Delta {b^2}} $$

Δ*L*, Δ*a*, and Δ*b* correspond to the difference in L*, a*, b* values before and after treatment, respectively, for each sample. The colorimetric experiments were repeated for three samples for each treatment.

The microstructure of the potato samples was observed under a stereomicroscope (Lumar, V12 SteREO Carl Zeiss; Carl Zeiss, Oberkochen, Germany) at a magnification of 45× and 80× 10 min after the treatment. The samples were stained with 0.1% toluidine blue O (TBO), to observe the cell walls (Obrien, Feder & McCully, 1964). We sectioned the potato cube and placed the sliced sections on a clean microscope slide. Then, we flooded the sections with an aqueous solution of 0.1% TBO for 1 min. The stain was removed gently from the potato surface by using a piece of filter paper and the samples were washed with water until there was no excess stain around the sample. Nine (3 × 3, three samples for each treatment and three different sections in each sample) sections were examined in each treatment.

Mean and standard deviations were calculated using SPSS 24.0 software (IBM, Armonk, NY, USA). *T*-ANOVA was used to detect significant differences between mean. Significance was set at *p* < 0.05 for the ANOVA matrix *F* value.

## Results and Discussion

The technology of an isochoric system is very simple relative to that of a comparable high-pressure freezing (hyperbaric) system. Unlike a hyperbaric freezing system, an isochoric system contains no moving parts and requires no power for continuous operation. There is also no concern with sealing the chamber around moving parts or deterioration of moving parts (Mikus et al., 2016; Rubinsky, Perez & Carlson, 2005). Figure 2A shows a schematic of the device. It is a rigid closed container designed to withstand the pressure. Figure 2B shows a photograph of the isochoric device used in this study. It is a capped cylinder made from a standard commercial stainless steel pressure vessel.

Control over the isochoric refrigeration process is also very simple. Figure 2 shows that we have used a pressure transducer connected to the vessel for control. In an isochoric refrigeration system, either only temperature or pressure need to be controlled. A two-phase system in a closed fixed volume is always at thermodynamic equilibrium. Therefore, either pressure or temperature completely specifies the system. In contrast, in a hyperbaric system, there is the need to control both temperature and pressure (Koch et al., 1996).

**Figure 3:**
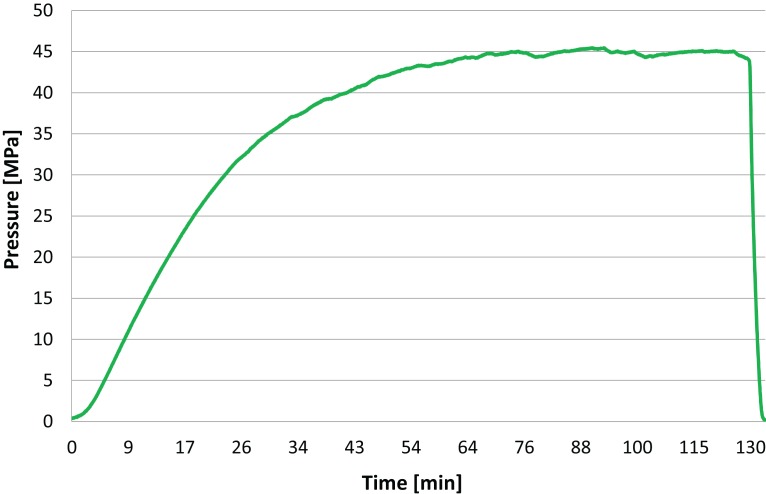
The pressure in the isochoric chamber was measured during the experiment with the pressure transducer in Fig. 2. This figure shows a typical pressure measurement over time in the isochoric system during the potato freezing experiment.

Figure 3 was obtained from measurements made with the pressure transducer in Fig. 2 during an isochoric freezing experiment. It shows a typical curve depicting the change in pressure with time during the isochoric refrigeration process in our experiments. The interesting aspect is that the pressure reaches steady state and stays at that value for over an hour, to the termination of the experiment. This demonstrates that the isochoric system has reached thermodynamic equilibrium. The time to reach steady state obviously depends on the thermal mass of the device and the heat transfer coefficient to the cooling bath. In all our experiments, the samples reached isochoric thermodynamic equilibrium, and our results represent the state of the treated material after it has reached thermodynamic equilibrium.

**Figure 4:**
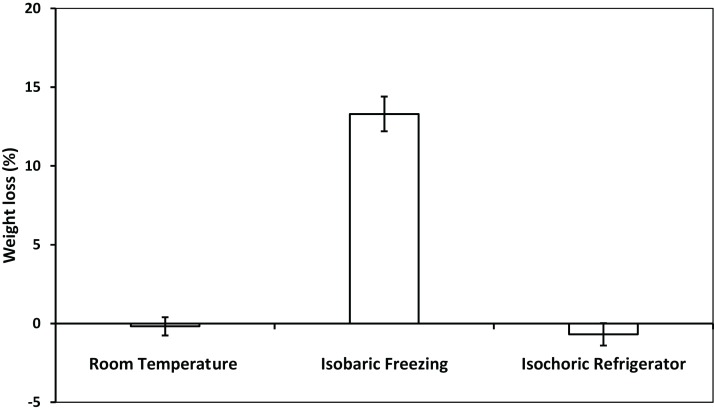
The weight loss of potato after room temperature preservation, isobaric freezing and isochoric refrigeration. The weight loss was obtained by comparing the weight of the samples before and after each treatment. The surface water on the sample was absorbed by filter papers before weighing. The weight loss of five samples were measured for each treatment. The error bars represent the standard deviation of five samples.

**Figure 5:**
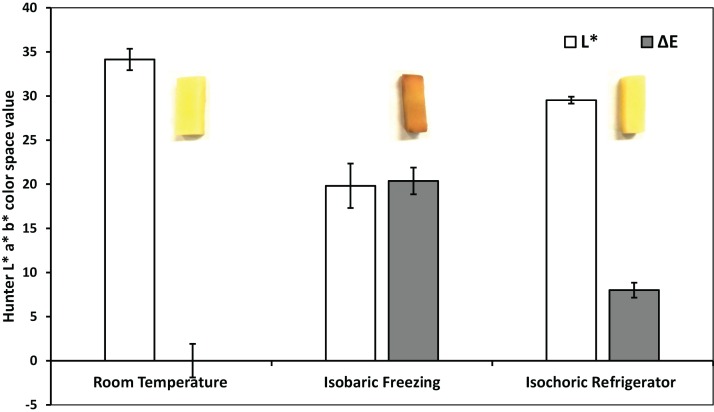
Colorimetric measurements—after room temperature preservation, isobaric freezing and isochoric freezing. Δ*E* (dark) and L* light data columns. L* represents the lightness of the color (L* = 0 yields black and L* = 100 indicates diffuse white), a* represents the redness and b* represents its yellowness in the color. Δ*E* is the total color difference which was calculated using $\Delta E = \sqrt {\Delta {L^2} + \Delta {a^2} + \Delta {b^2}} $, where Δ*L*, Δ*a*, and Δ*b* are the differences in L*, a*, b* values before and after treatment, respectively, in each sample. The colorimetric experiments were repeated on three samples for each treatment. The values on left are for both, Δ*E* and L*. The error bars represent the standard deviation of three samples. Inserts, macroscopic photographs of the potato samples.

**Figure 6:**
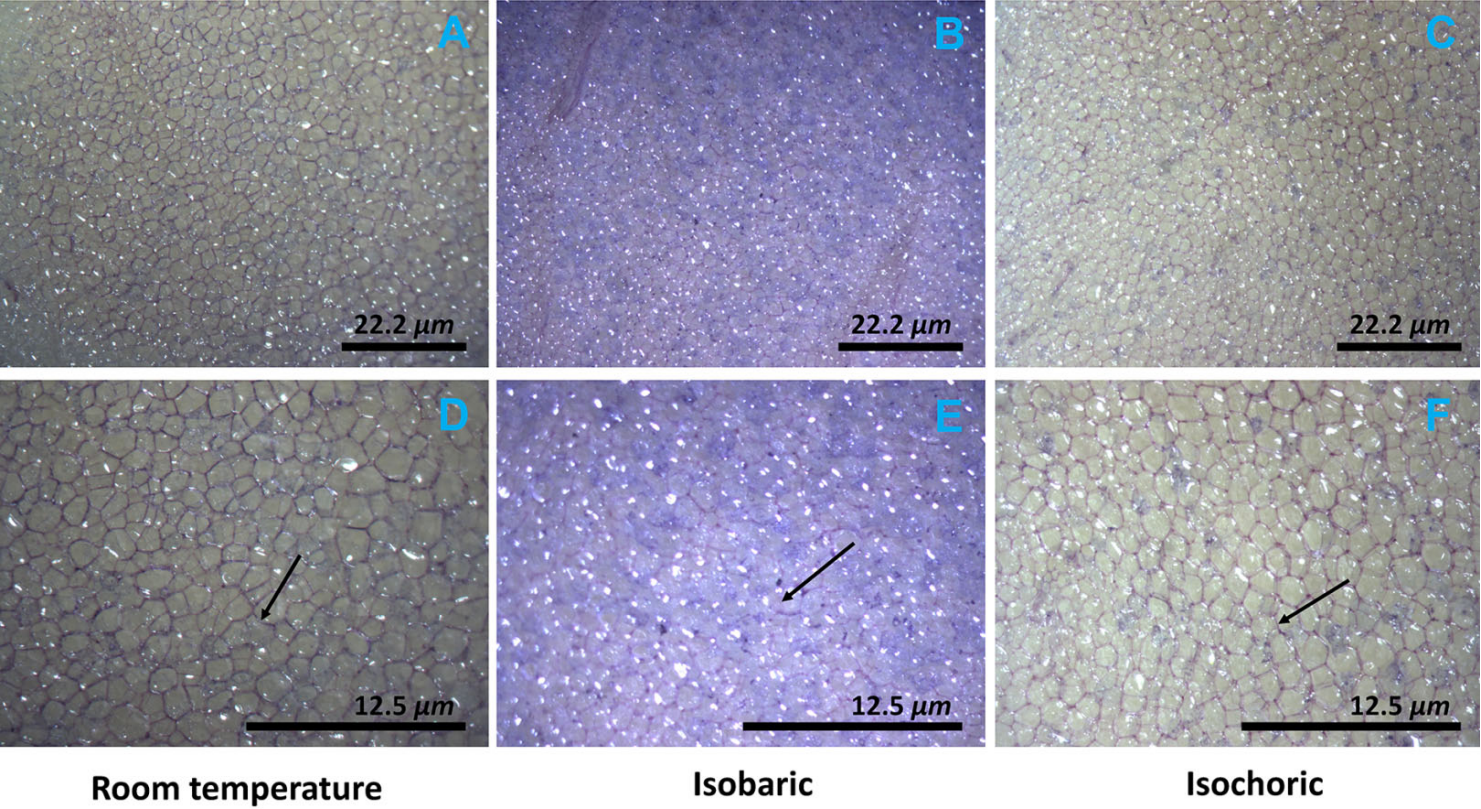
Microscopic photographs of the potato after isochoric refrigeration and isobaric freezing. The arrow points to a typical cell wall. Note the color in the micrographs. The microstructure of potatoes was observed by stereomicroscope (Lumar, V12 Stereo Zeiss) within 10 min after the treatment. The samples were stained by 0.11% toluidine blue O for 1 min to observe the cell walls of potato. Nine (3 × 3, three samples of each treatment and three different sections in each sample) sections were examined in each treatment. Top row (A–C) ×45, scale bar 22.2 μm; bottom row (D–F) ×80, scale bar 12.5 μm.

Figures 4 and 5 compare, respectively, the weight loss and color change after 2 h of: (a) freezing to −5 °C in an isochoric system; (b) freezing to −5 °C in isobaric conditions; and (c) storage in an isotonic sucrose solution at room temperature. Figure 6 describes the microscopic micrographs that provide an explanation for the mechanisms involved.

Weight loss during storage is of concern to the food industry. It occurs during preservation of all foods, including potatoes (Wang, Brandt & Olsen, 2016). Frozen storage is particularly detrimental as it leads to substantial weight loss (Campañone, Salvadori & Mascheroni, 2001; Koch et al., 1996). Figure 4 shows a comparison between the change in weight of the potato samples after 2 h in a 9.09% w/w sucrose solution at: room temperature, −5 °C in isobaric condition, and −5 °C in isochoric condition. The figure shows that there is no statistically significant change in weight either after 2 h at room temperature or during freezing at −5 °C in isochoric conditions (*p* > 0.05). In contrast, freezing at −5 °C in isobaric conditions resulted in a weight loss of 13.1 ± 1.1%. The weight loss with isobaric freezing observed here is consistent with findings of many other studies (Koch et al., 1996). To the best of our knowledge, the fact that there was no weight loss after isochoric freezing at temperatures lower than 0 °C, is unique to isochoric refrigeration.

Browning in raw fruits, vegetables and their processed products is a major problem in the food industry and is believed to be one of the main causes of quality loss during post-harvest handling and processing. The browning reaction in the potato is an important area of research in the food industry and was studied for well over half a century (Schwimmer & Makower, 1954). It results from the oxidation of phenolic compounds under the action of an enzyme called polyphenol oxidase (PPO, phenolase). In the presence of oxygen from air, the enzyme catalyzes the first steps in the biochemical conversion of iron-containing phenolics, found in the potato, to produce quinone which undergo further polymerization to yield dark insoluble polymers referred to as “melanin.” Browning and the formation of melanin occur in the potato when the PPO enzyme is released through damaged cell membranes. Figure 5 shows the color difference between the samples kept at room temperature, those frozen to −5 °C in isobaric conditions and those frozen to −5 °C in isochoric conditions. For each case, the figure shows a typical photograph of the sample, the total color change $\Delta E = \sqrt {\Delta {L^2} + \Delta {a^2} + \Delta {b^2}} $, and the changed in lightness, L*. Obviously, browning is substantially reduced in isochoric refrigeration relative to isobaric freezing to the same temperature; which is another potentially important attribute of isochoric refrigeration.

Figure 6 shows microscopic images of the treated samples and the effects of isochoric refrigeration. The micrographs show the appearance of the samples after staining with the toluidine blue stain, at two magnifications, ×45 (top row) and ×80 (bottom row). In analyzing the micrographs, it is important to realize that toluidine stains the cell walls in plants as well as the starch (Obrien, Feder & McCully, 1964). The arrow points to the cell wall. It is obvious that the cell walls in the room temperature sample and the isochoric −5 °C samples are intact and encircle the cells. Furthermore, the tissue is translucent. In contrast, the arrow in Fig. 6 shows that in the isobaric frozen sample, the cell walls are impaired. Also, important is the observation that the toluidine has stained the entire volume of the potato. This suggests that the cell membrane was breached and the intracellular starch has become accessible to the stain throughout the sample. In contrast, there is no staining of starch either in the room temperature stored sample or in the isochoric preserved sample. The fact that the intracellular content was released after isobaric freezing explains both the changes in weight and the browning of the isobaric preserved samples, in relation to the room temperature preserved samples and the isochoric preserved samples in Figs. 4 and 5.

The mechanisms of damage during isobaric freezing were discussed in the “Introduction” (Reid, 1996). According to Fig. 1A, only 25% of the water is frozen in an isochoric system at −5 °C. Obviously, in the isochoric system in our particular design, ice is produced distant from the biological material and there is no ice in the preserved biological material. Therefore, the mechanism of cell damage by freezing is eliminated. In contrast, from conservation of mass and Fig. 1B, it is possible to estimate that in an isobaric system at −5 °C, 85% of the volume is frozen, and the freezing engulfs the potato. With respect to solute concentration, our analysis and experiments (Fig. 1A) show that when a solution is frozen under isobaric atmospheric conditions to −5 °C, the osmolality increases to 3.5 Osm (Rubinsky, Perez & Carlson, 2005). In contrast, when the solution is frozen to −5 °C under isochoric conditions, the osmolality of the unfrozen milieu composition is lower by a factor of seven from that in an isobaric system (Rubinsky, Perez & Carlson, 2005). The increase in extracellular osmolality during isobaric freezing may be another factor contributing to the detected weight loss, because of the dehydration effect.

It should be noticed, though, that in isochoric refrigeration the pressure increases, while in an isobaric system the pressure remains constant. Obviously, this is a potential mechanism of cell damage during isochoric freezing that does not exist in isobaric freezing. However, the increase in pressure in this experiment is hydrostatic and mild. Experiments have shown that even whole livers can survive the pressures in our isochoric experiments conditions (Takahashi et al., 2001). This should explain why the cell membrane is intact and the intracellular content is maintained in isochoric refrigeration. The integrity of the cell membrane and the isosmotic composition of the intracellular milieu and the extracellular milieu during isochoric refrigeration is the reason why there is no weight loss or substantial browning during isochoric refrigeration to −5 °C as shown in Figs. 4 and 5. In contrast, the breaching of the cell membrane and the hyperosmotic extracellular concentration in isobaric freezing to −5 °C results in weight loss to the extracellular milieu and browning of the intracellular. It is possible to draw a practical conclusion from these experiments. Metabolic analysis predicts that lowering the storage temperature of a meat product from 4 to −5 °C will reduce metabolism by a factor of between two and three. This suggests that a minor change in storage conditions, from 4 °C isobaric to −5 °C isochoric could double the storage time of a product with minimal effect on the quality. Obviously, this is an extrapolation that needs to be examined.

In summary, this is a first experimental study on the feasibility of isochoric refrigeration of a food product at subfreezing temperatures. While obviously much more research must be done on this technology, it is evident that a food product, such as the potato, can be preserved at −5 °C in isochoric conditions without the deleterious effects of isobaric atmospheric freezing to −5 °C, i.e., weight loss and browning. It should be emphasized that we have focused here on the damage due to freezing and not storage damage. A further study on storage damage is also needed. In addition, our laboratory is equipped for mechanical engineering work and does not have the devices necessary for chemical and nutritional studies on food quality deterioration, such as changes in vitamin C. There is no doubt that much more research is needed on the effects of storage, on chemical changes, and of course a more extensive study over the entire range of temperatures to the triple point. Nevertheless, the main value of this study is that it introduces a possible new method of food storage, with some apparent potential.

## Supplemental Information

10.7717/peerj.3322/supp-1 Supplemental Information 1 Raw Figure 1. Potato fresh 45x–no TBO (scale bar 22.2 *μm*).

10.7717/peerj.3322/supp-2 Supplemental Information 2 Raw Figure 2. Potato fresh 45x with TBO (scale bar 22.2 *μm*).

10.7717/peerj.3322/supp-3 Supplemental Information 3 Raw Figure 3. Potato fresh 80x–no TBO (scale bar 12.5 *μm*).

10.7717/peerj.3322/supp-4 Supplemental Information 4 Raw Figure 4. Potato fresh 80x–TBO (scale bar 12.5 *μm*).

10.7717/peerj.3322/supp-5 Supplemental Information 5 Raw Figure 5. Potato isobaric 45x–no TBO (scale bar 22.2 *μm*).

10.7717/peerj.3322/supp-6 Supplemental Information 6 Raw Figure 6. Potato isobaric 45x–TBO (scale bar 22.2 *μm*).

10.7717/peerj.3322/supp-7 Supplemental Information 7 Raw Figure 7. Potato isobaric 80x–no TBO (scale bar 12.5 *μm*).

10.7717/peerj.3322/supp-8 Supplemental Information 8 Raw Figure 8. Potato isobaric 80x–TBO (scale bar 12.5 *μm*).

10.7717/peerj.3322/supp-9 Supplemental Information 9 Raw Figure 9. Potato isochoric 45x_1–no TBO(scale bar 22.2 *μm*).

10.7717/peerj.3322/supp-10 Supplemental Information 10 Raw Figure 10. Potato isochoric 45x_2–no TBO (scale bar 22.2 *μm*).

10.7717/peerj.3322/supp-11 Supplemental Information 11 Raw Figure 11. Potato isochoric 45x_1–TBO (scale bar 22.2 *μm*).

10.7717/peerj.3322/supp-12 Supplemental Information 12 Raw Figure 12. Potato isochoric 45x_2–TBO (scale bar 22.2 *μm*).

10.7717/peerj.3322/supp-13 Supplemental Information 13 Raw Figure 13. Potato isochoric 80x–no TBO (scale bar 12.5 *μm*).

10.7717/peerj.3322/supp-14 Supplemental Information 14 Raw Figure 14. Potato isochoric 80x_1–TBO (scale bar 12.5 *μm*).

10.7717/peerj.3322/supp-15 Supplemental Information 15 Raw Figure 15. Potato isochoric 80x_2–TBO (scale bar 12.5 *μm*).

## Additional Information and Declarations

### Data Availability

The following information was supplied regarding data availability: The raw data has been supplied as Supplemental Dataset Files.
