# Supplementary figures and images for: A comparison of freezing-damage during isochoric and isobaric freezing of the potato
Source: PeerJ. 2017 May 18;5:e3322. doi: 10.7717/peerj.3322 (PMC5438586; doi:10.7717/peerj.3322)

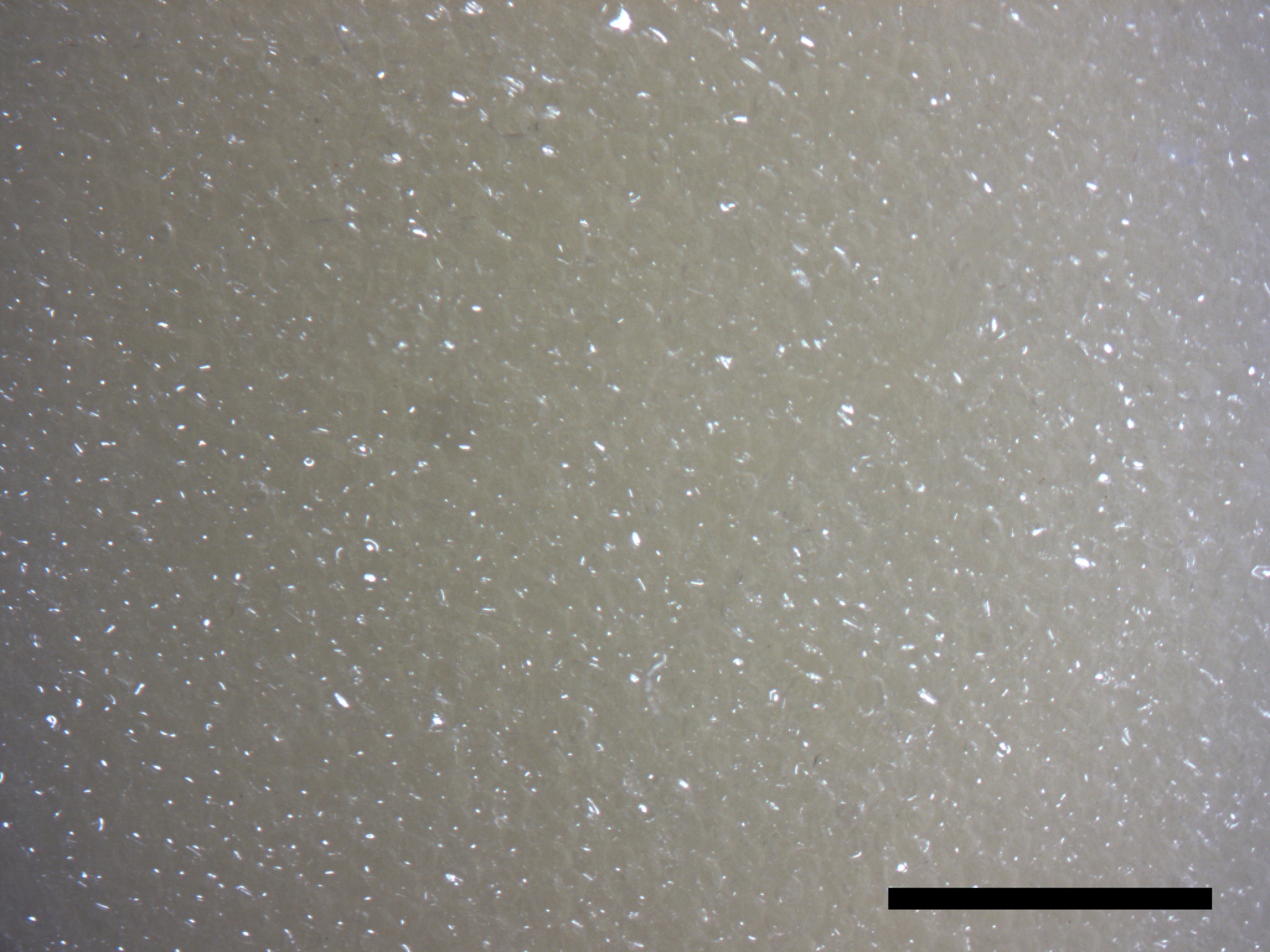

Supplement: Supplemental Information 1 — Potato fresh 45x–no TBO (scale bar 22.2 μm). [file peerj-05-3322-s001.jpg]

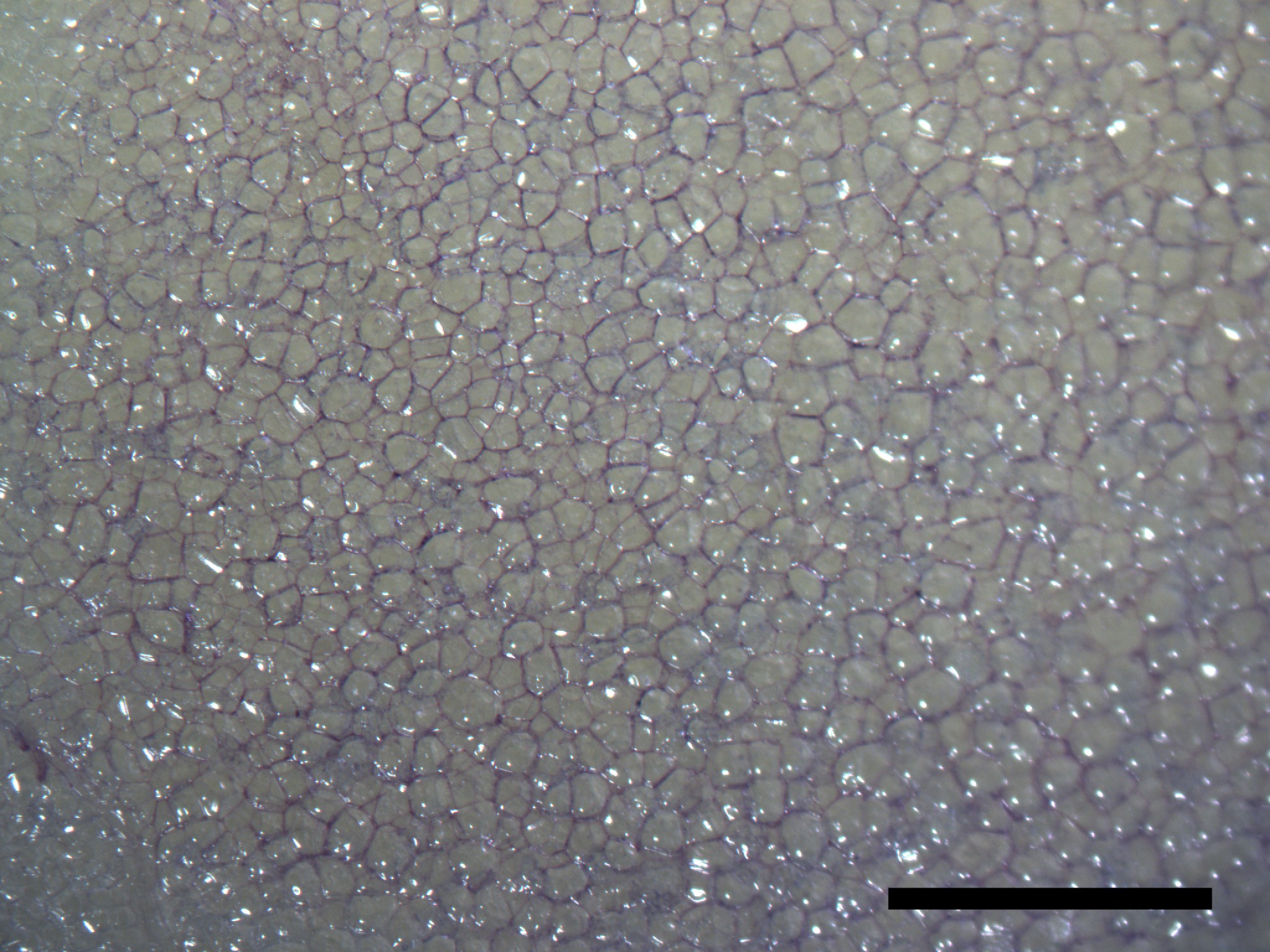

Supplement: Supplemental Information 2 — Potato fresh 45x with TBO (scale bar 22.2 μm). [file peerj-05-3322-s002.jpg]

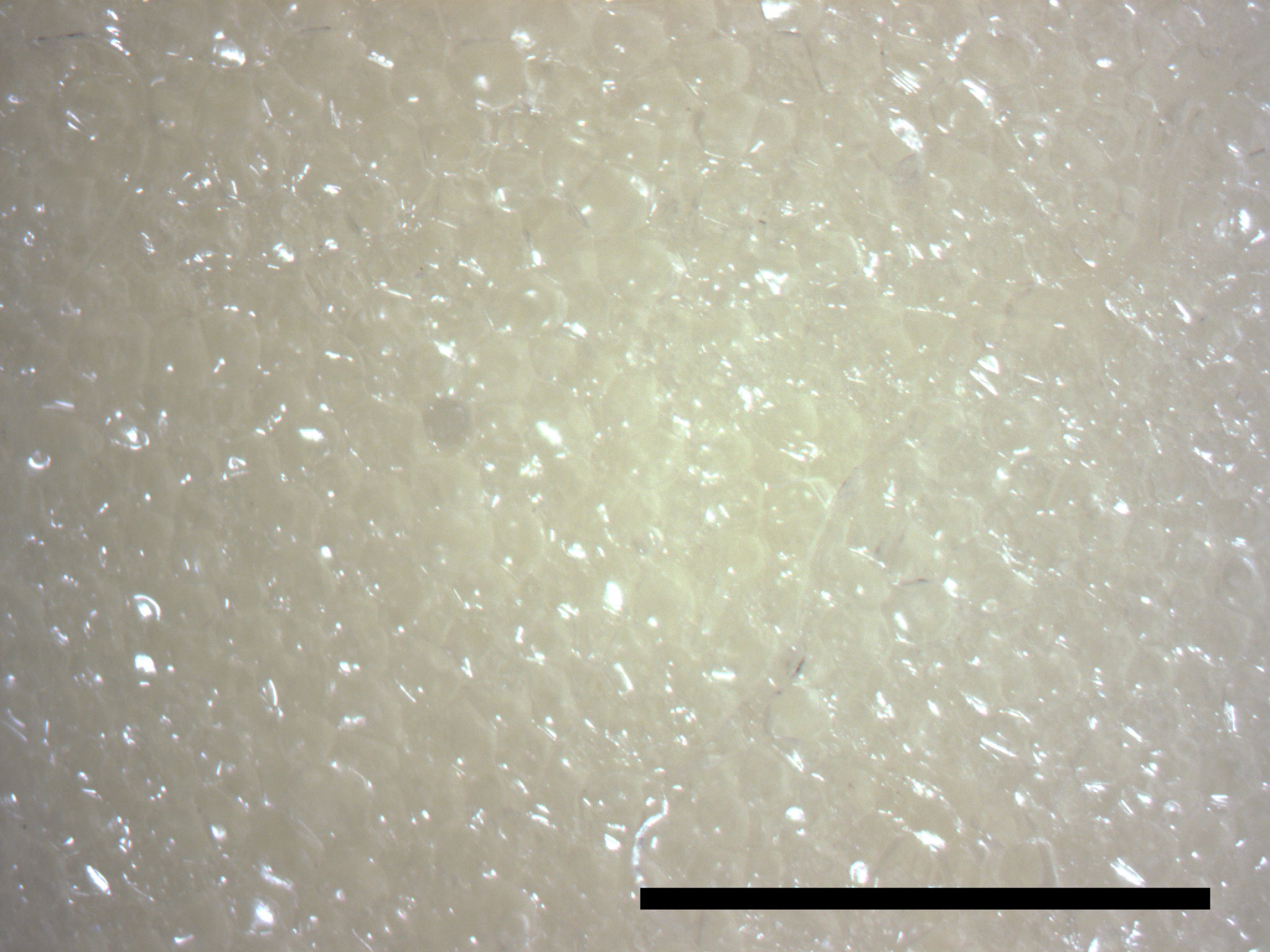

Supplement: Supplemental Information 3 — Potato fresh 80x–no TBO (scale bar 12.5 μm). [file peerj-05-3322-s003.jpg]

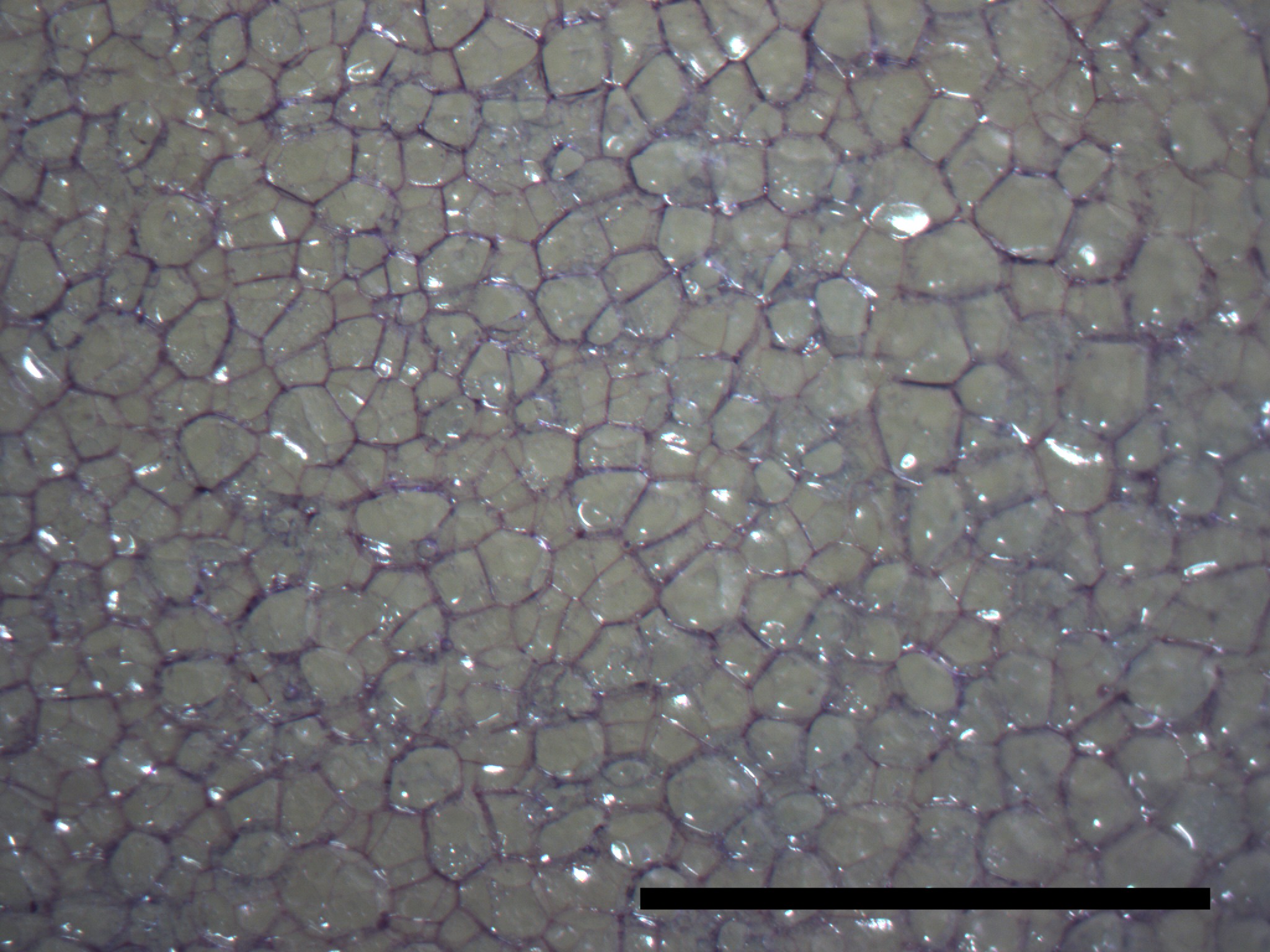

Supplement: Supplemental Information 4 — Potato fresh 80x–TBO (scale bar 12.5 μm). [file peerj-05-3322-s004.jpg]

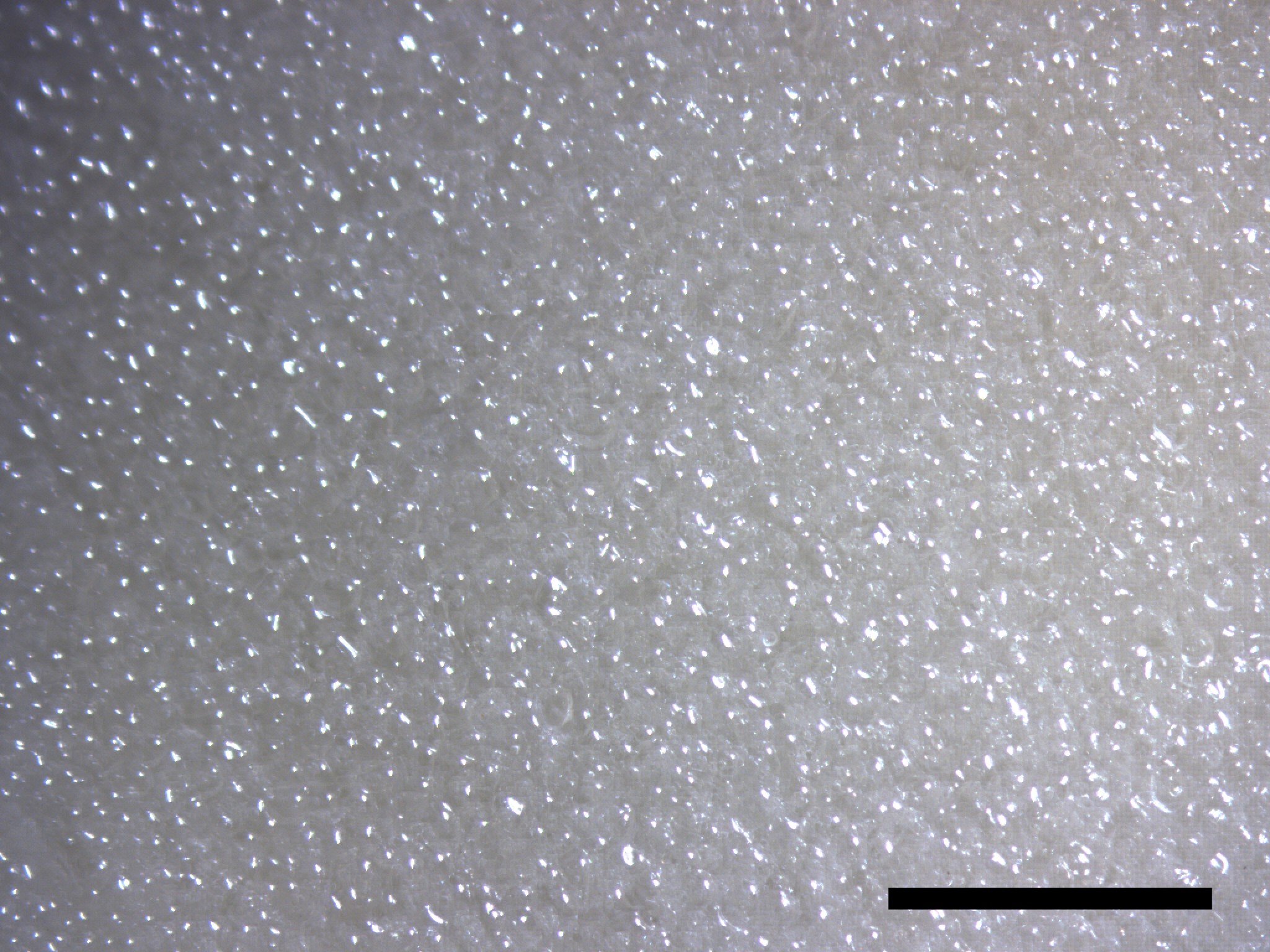

Supplement: Supplemental Information 5 — Potato isobaric 45x–no TBO (scale bar 22.2 μm). [file peerj-05-3322-s005.jpg]

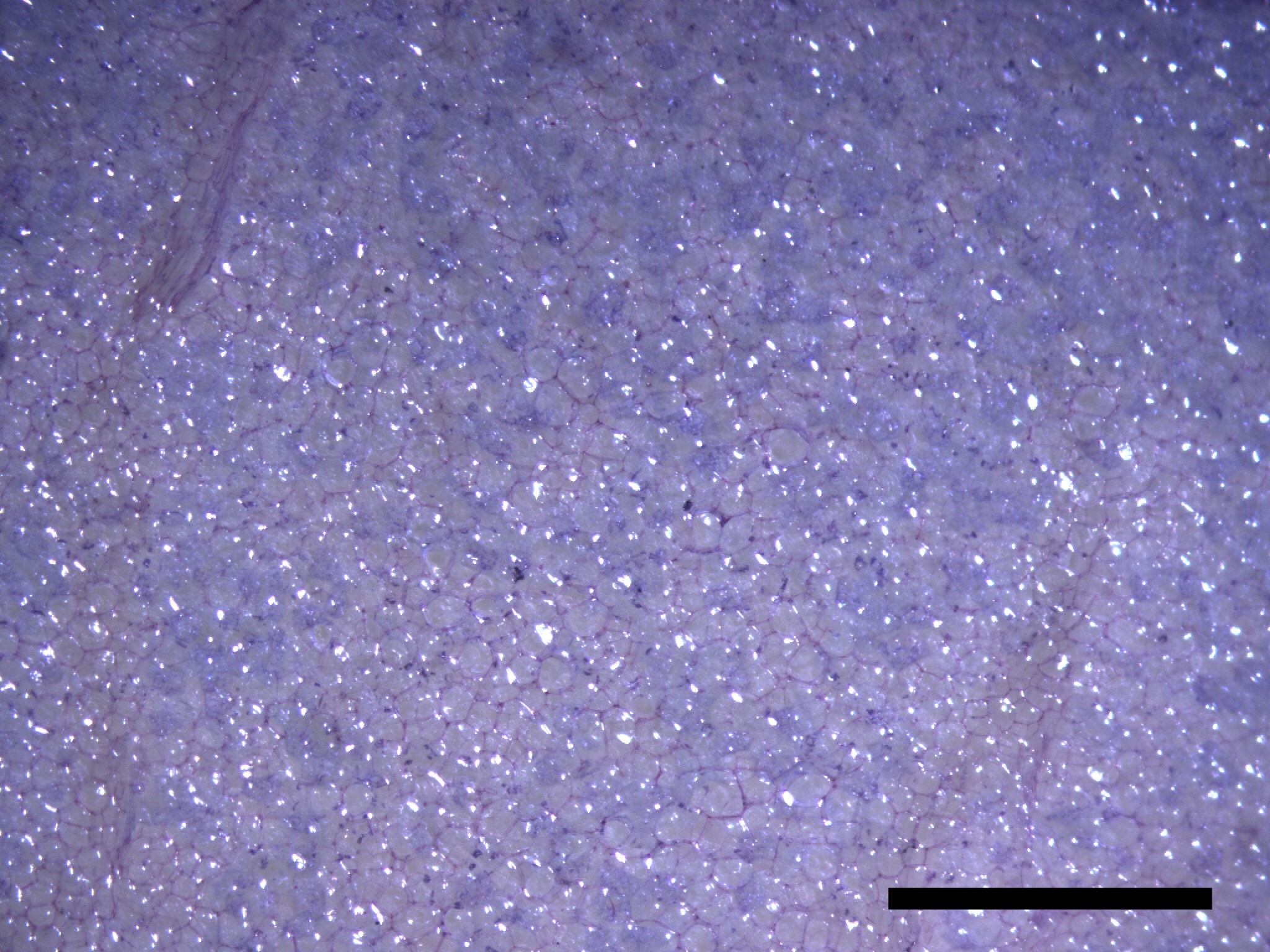

Supplement: Supplemental Information 6 — Potato isobaric 45x–TBO (scale bar 22.2 μm). [file peerj-05-3322-s006.jpg]

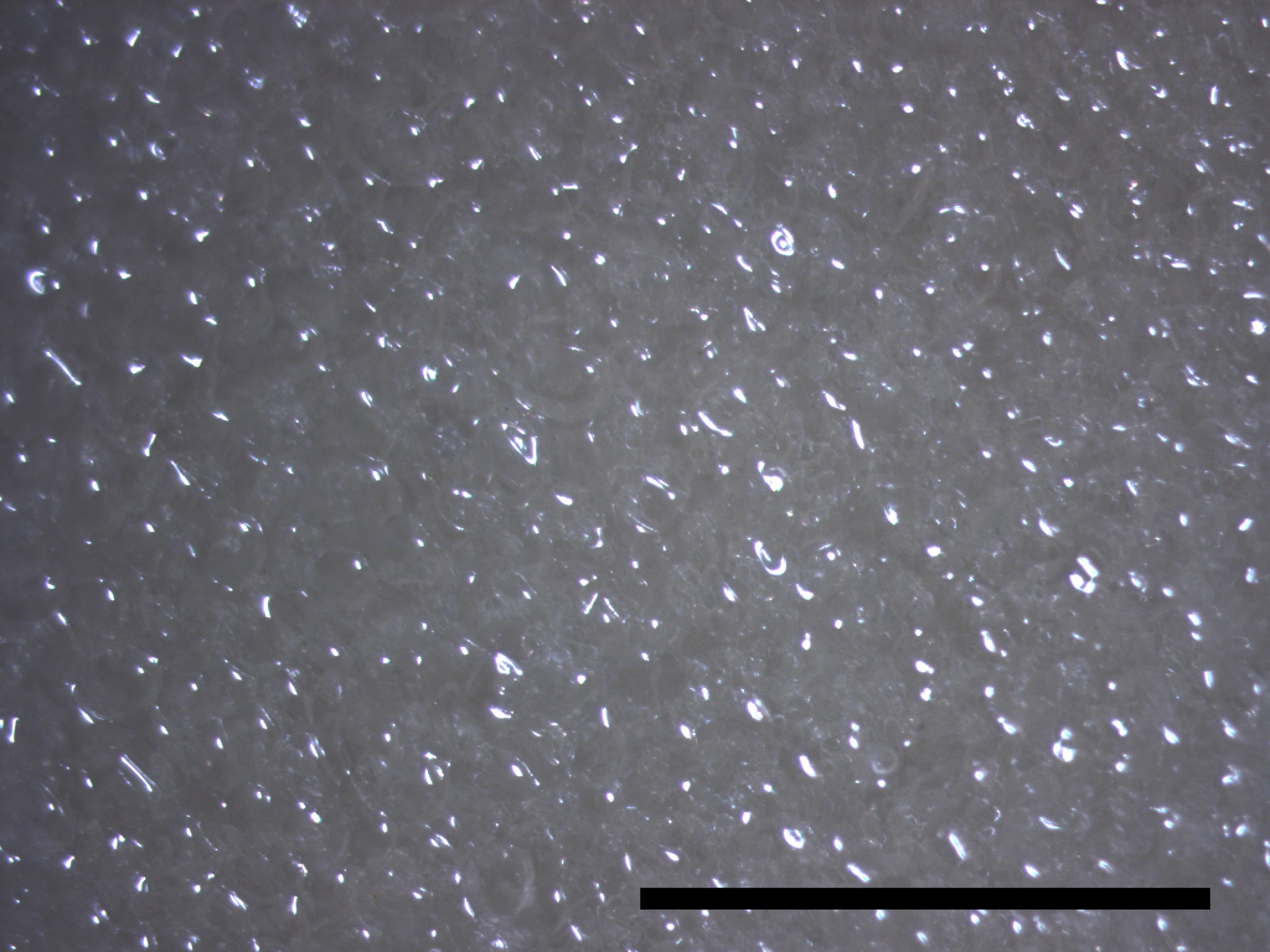

Supplement: Supplemental Information 7 — Potato isobaric 80x–no TBO (scale bar 12.5 μm). [file peerj-05-3322-s007.jpg]

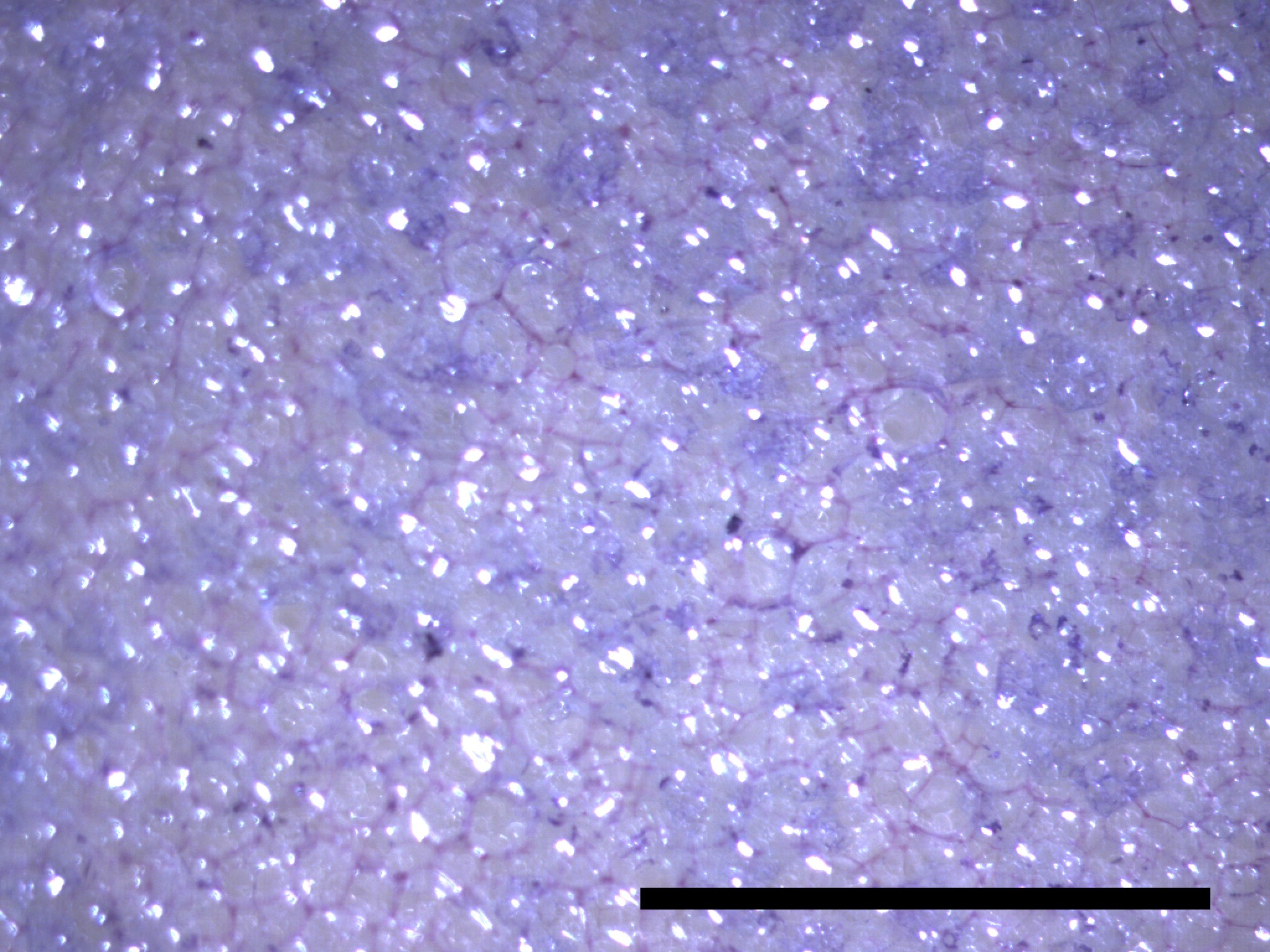

Supplement: Supplemental Information 8 — Potato isobaric 80x–TBO (scale bar 12.5 μm). [file peerj-05-3322-s008.jpg]

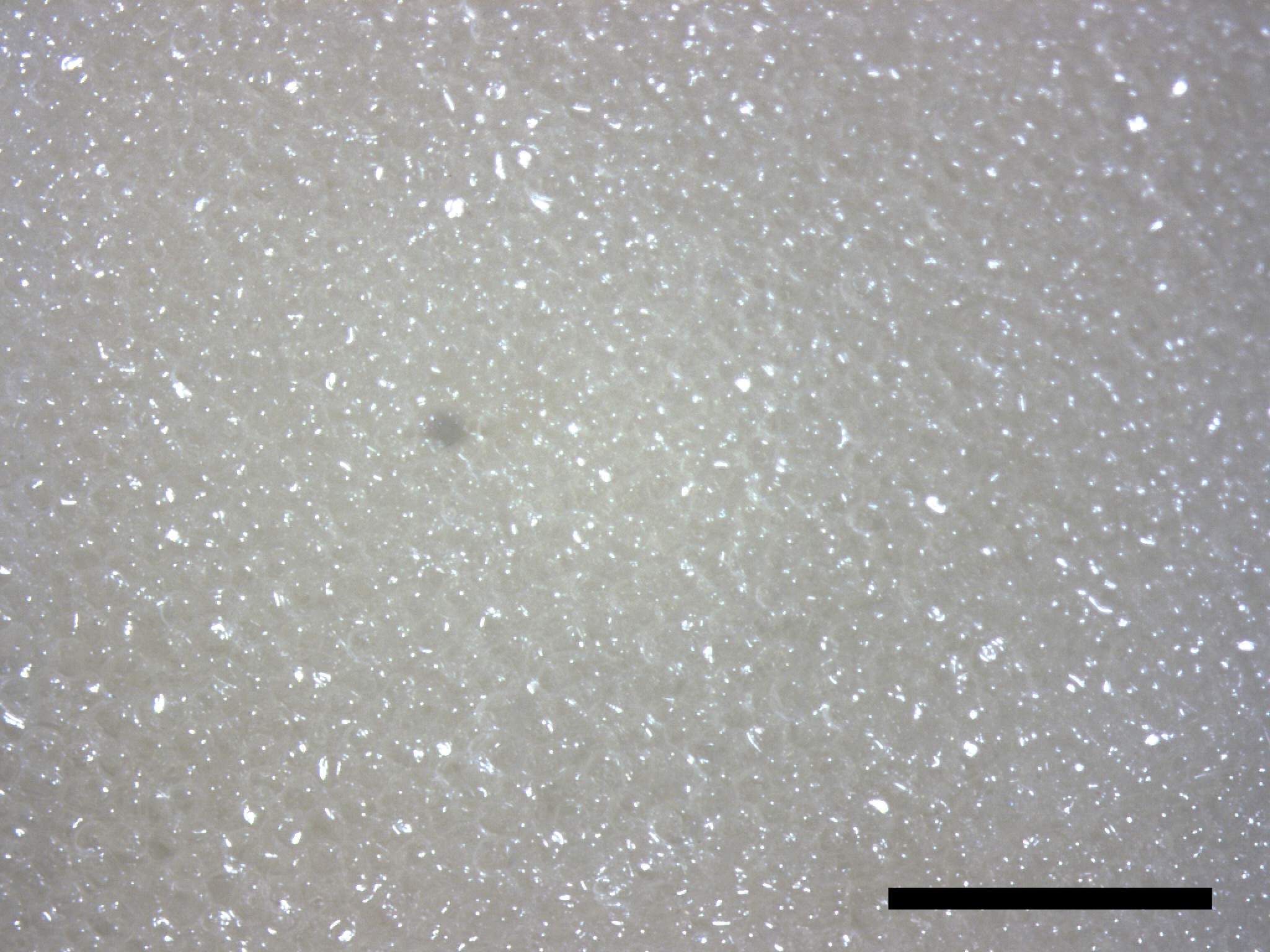

Supplement: Supplemental Information 9 — Potato isochoric 45x_1–no TBO(scale bar 22.2 μm). [file peerj-05-3322-s009.jpg]

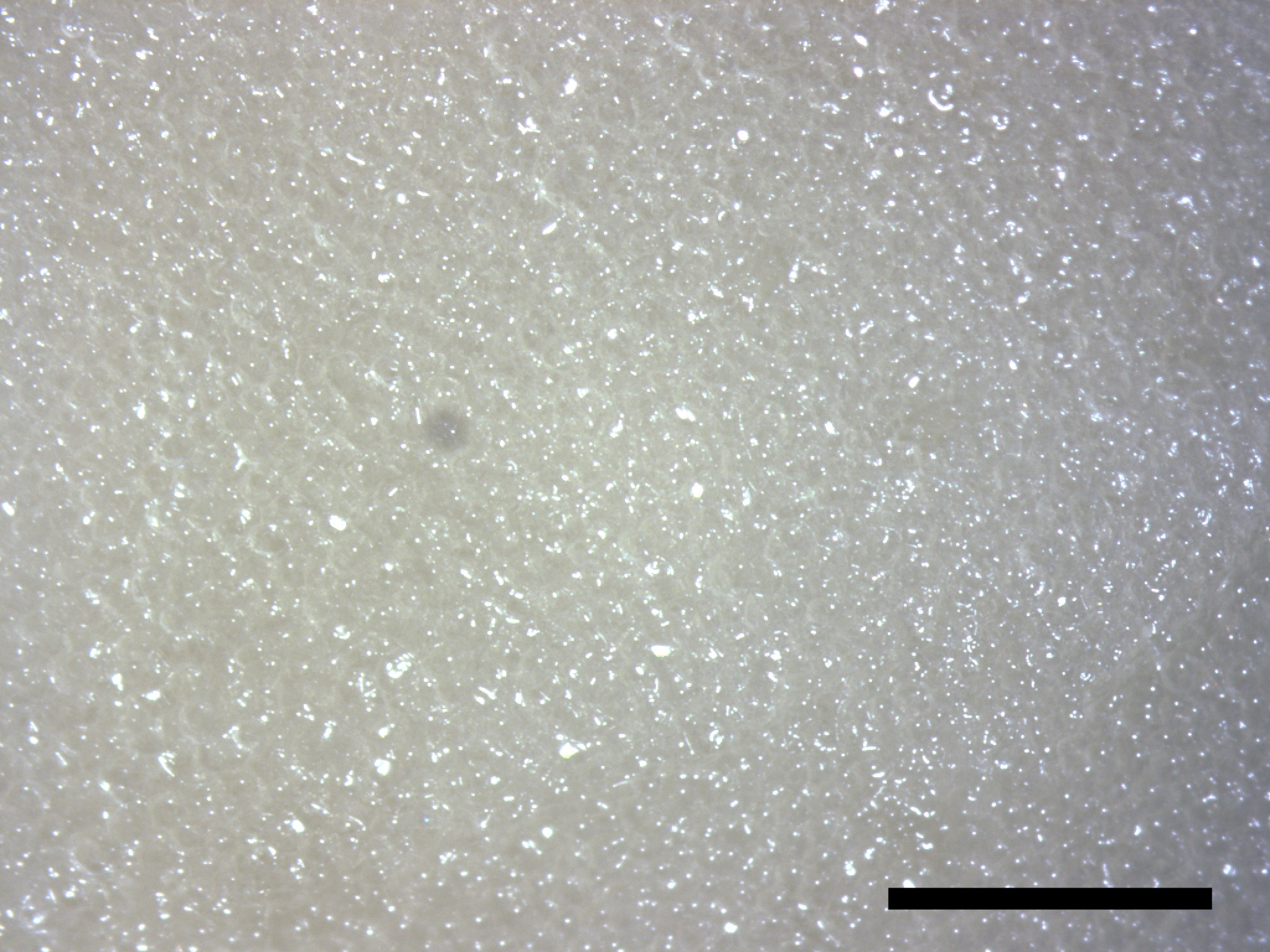

Supplement: Supplemental Information 10 — Potato isochoric 45x_2–no TBO (scale bar 22.2 μm). [file peerj-05-3322-s010.jpg]

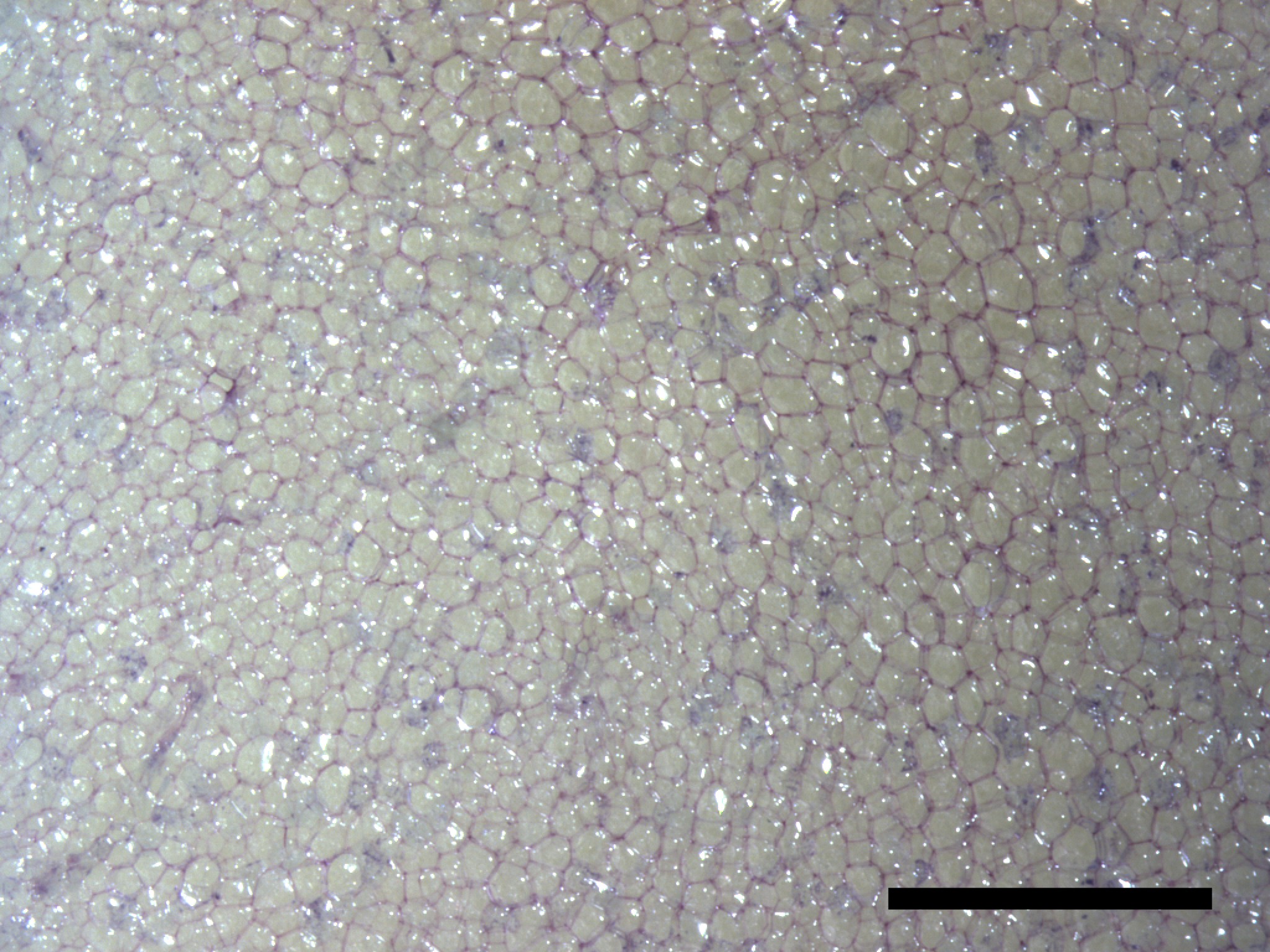

Supplement: Supplemental Information 11 — Potato isochoric 45x_1–TBO (scale bar 22.2 μm). [file peerj-05-3322-s011.jpg]

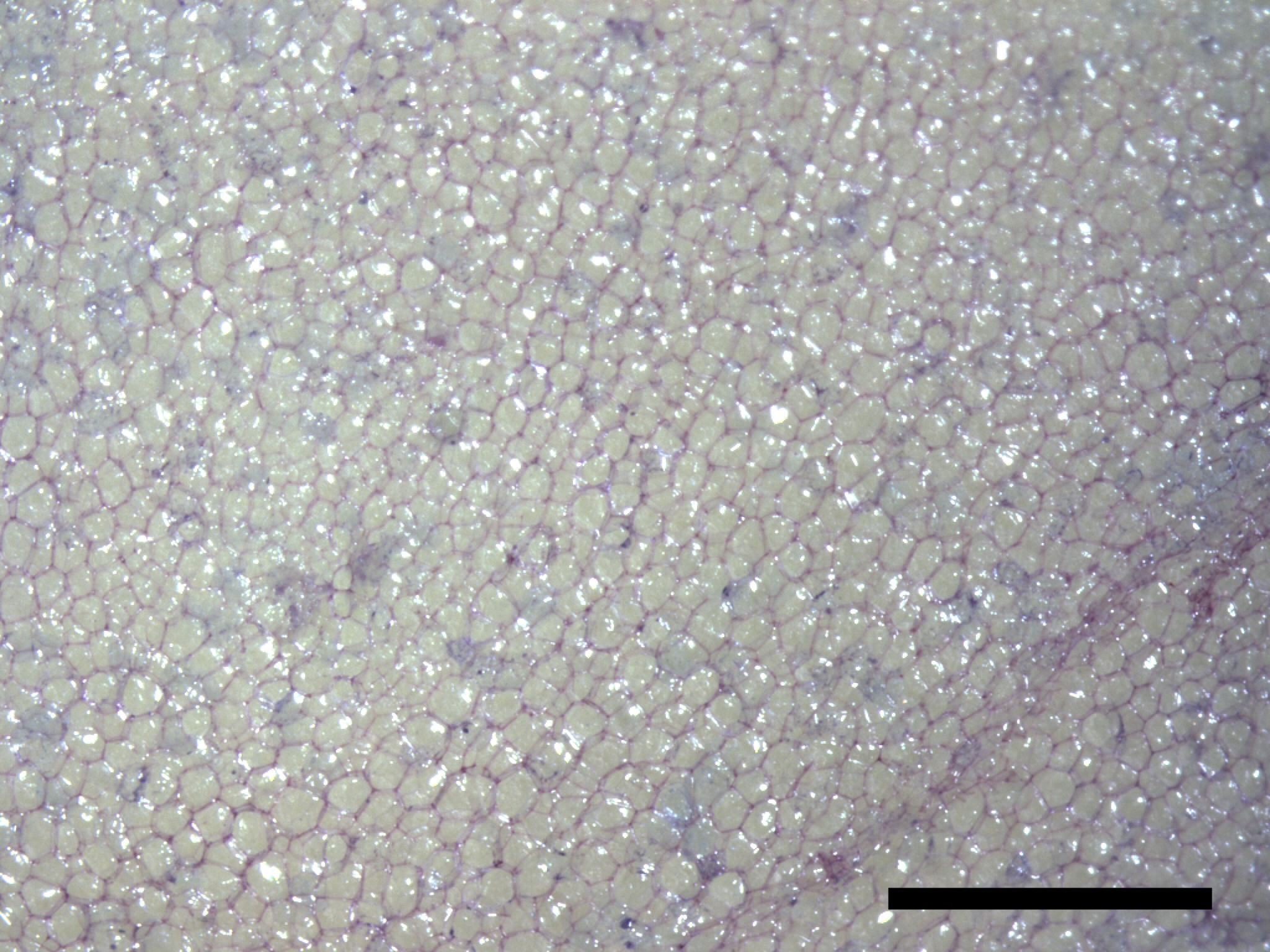

Supplement: Supplemental Information 12 — Potato isochoric 45x_2–TBO (scale bar 22.2 μm). [file peerj-05-3322-s012.jpg]

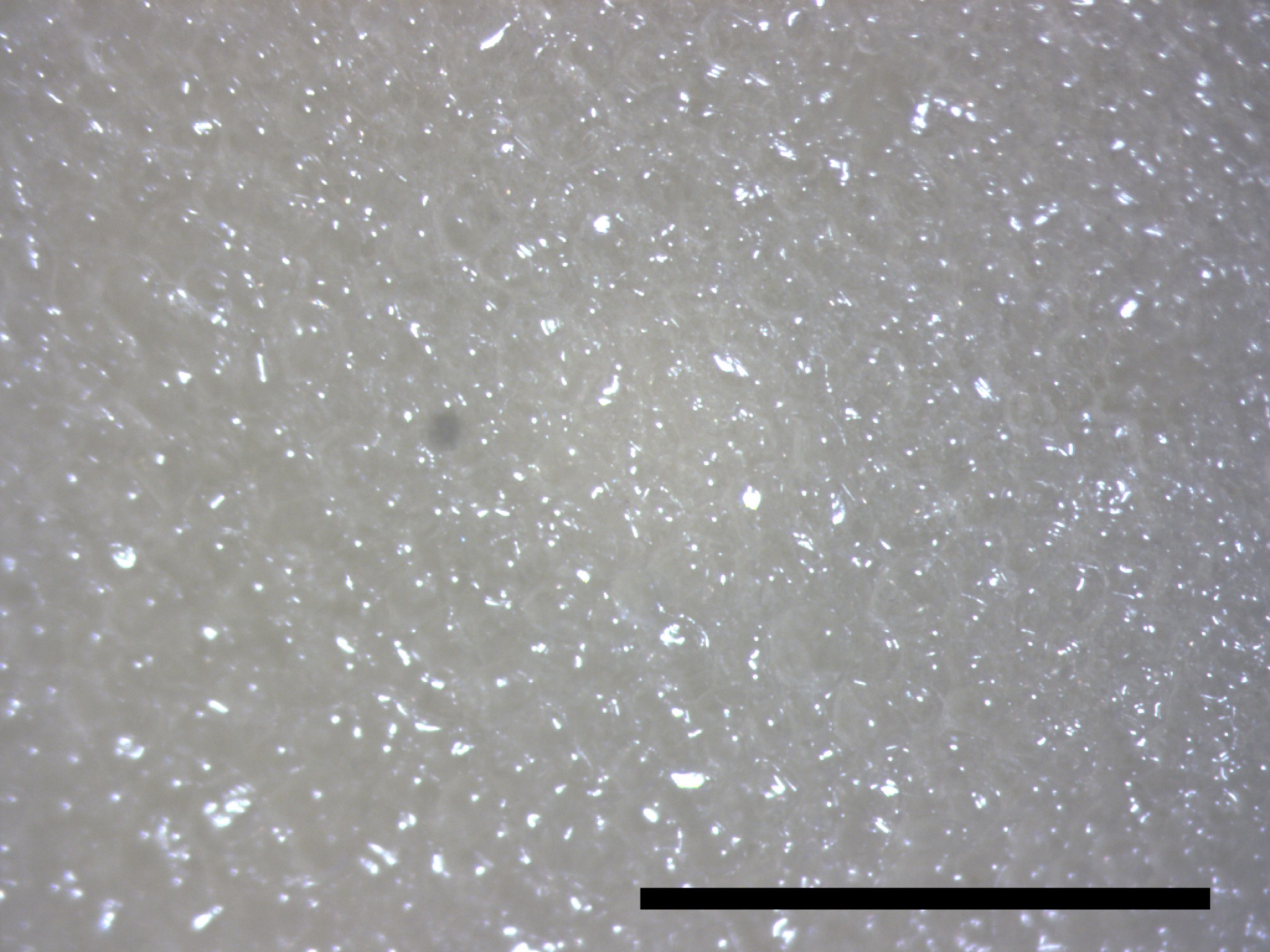

Supplement: Supplemental Information 13 — Potato isochoric 80x–no TBO (scale bar 12.5 μm). [file peerj-05-3322-s013.jpg]

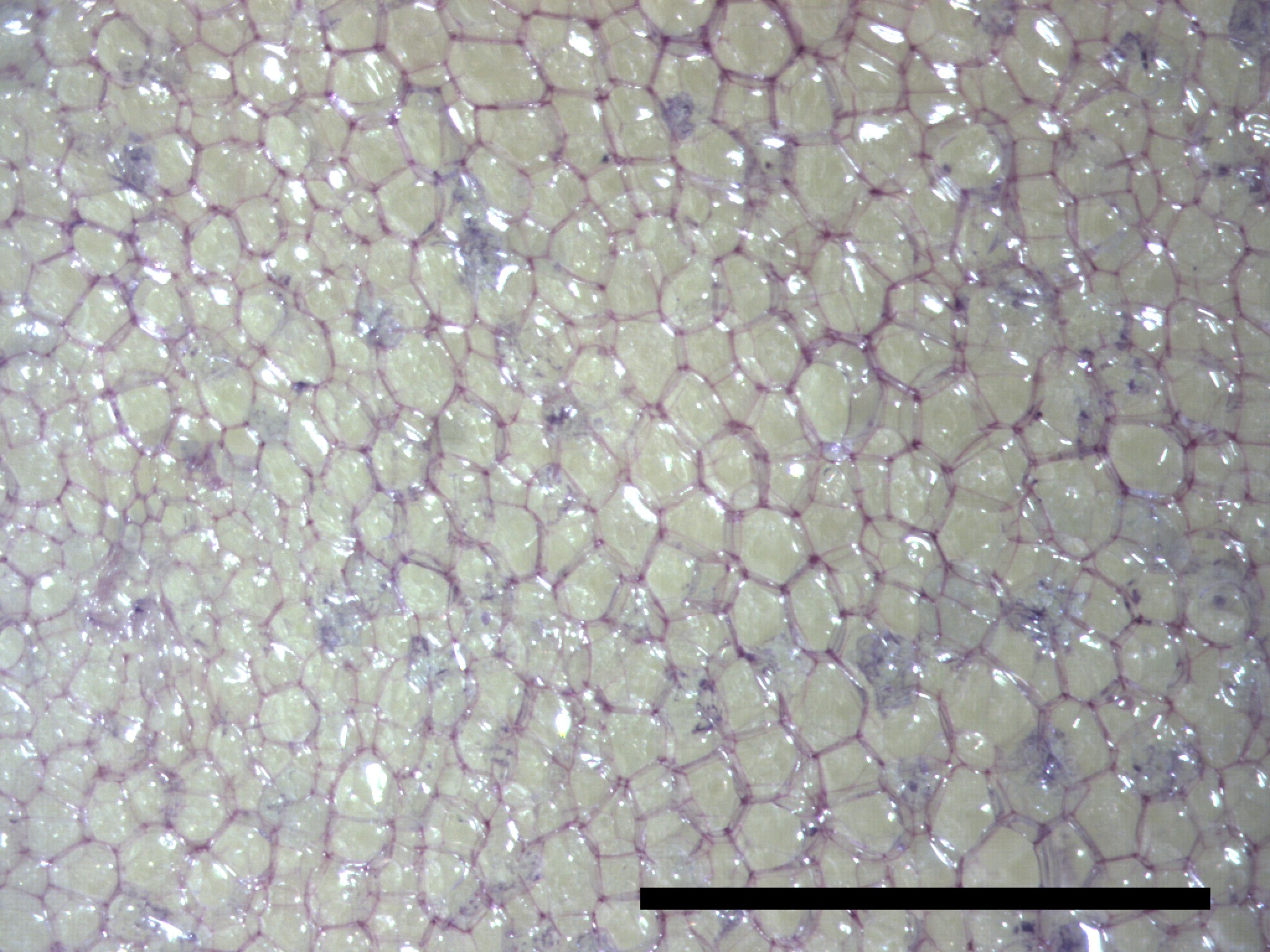

Supplement: Supplemental Information 14 — Potato isochoric 80x_1–TBO (scale bar 12.5 μm). [file peerj-05-3322-s014.jpg]

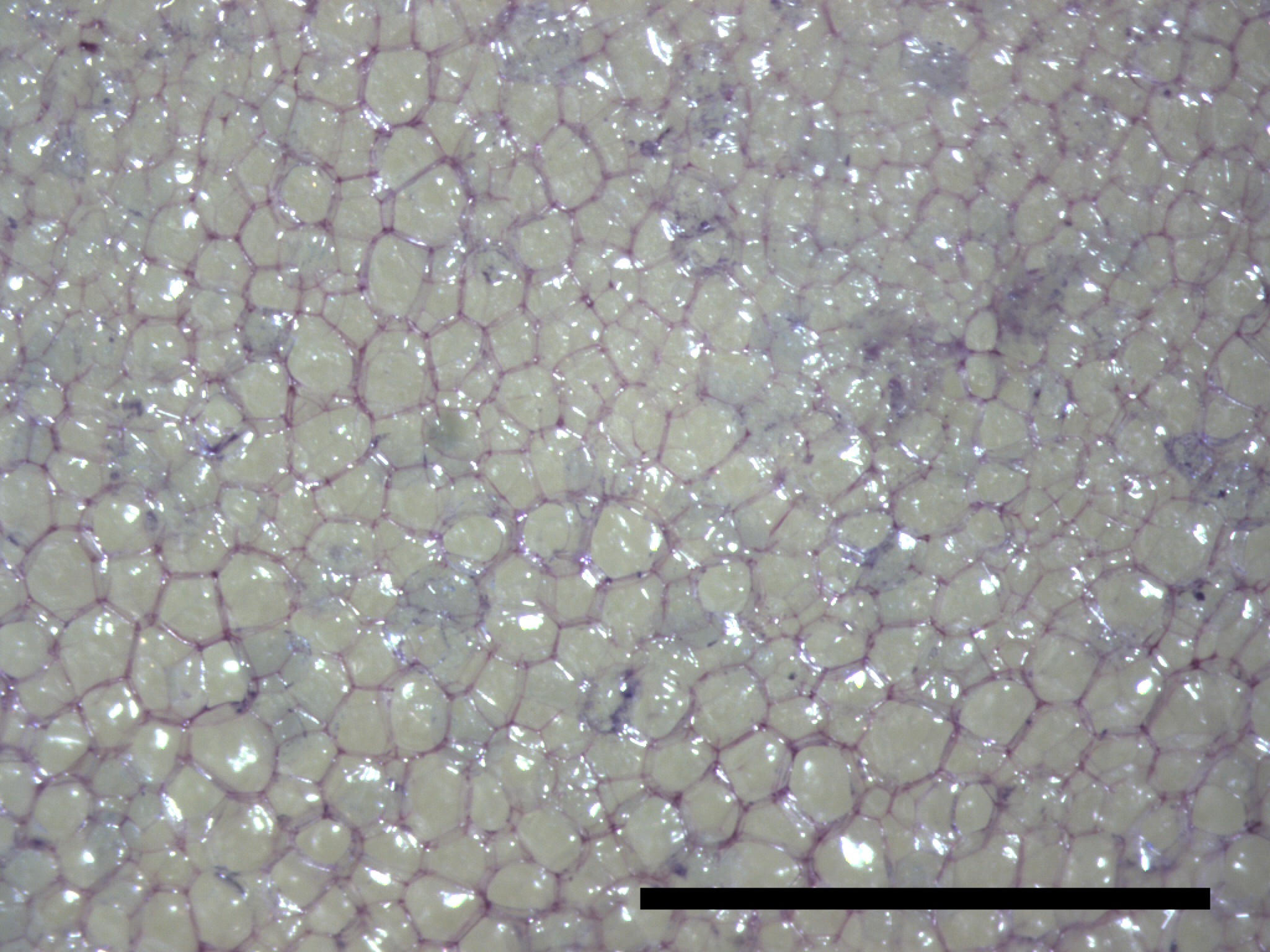

Supplement: Supplemental Information 15 — Potato isochoric 80x_2–TBO (scale bar 12.5 μm). [file peerj-05-3322-s015.jpg]
